# Targeted Over-Expression of Glutamate Transporter 1 (GLT-1) Reduces Ischemic Brain Injury in a Rat Model of Stroke

**DOI:** 10.1371/journal.pone.0022135

**Published:** 2011-08-10

**Authors:** Brandon K. Harvey, Mikko Airavaara, Jason Hinzman, Emily M. Wires, Matthew J. Chiocco, Douglas B. Howard, Hui Shen, Greg Gerhardt, Barry J. Hoffer, Yun Wang

**Affiliations:** 1 Intramural Research Program, National Institute on Drug Abuse, National Institutes of Health, Baltimore, Maryland, United States of America; 2 Department of Anatomy and Neurobiology, Center for Microelectrode Technology, University of Kentucky College of Medicine, Lexington, Kentucky, United States of America; Johns Hopkins, United States of America

## Abstract

Following the onset of an ischemic brain injury, the excitatory neurotransmitter glutamate is released. The excitotoxic effects of glutamate are a major contributor to the pathogenesis of a stroke. The aim of this study was to examine if overexpression of a glutamate transporter (GLT-1) reduces ischemic brain injury in a rat model of stroke. We generated an adeno-associated viral (AAV) vector expressing the rat GLT-1 cDNA (AAV-GLT1). Functional expression of AAV-GLT1 was confirmed by increased glutamate clearance rate in non-stroke rat brain as measured by in vivo amperometry. AAV-GLT1 was injected into future cortical region of infarction 3 weeks prior to 60 min middle cerebral artery occlusion (MCAo). Tissue damage was assessed at one and two days after MCAo using TUNEL and TTC staining, respectively. Behavioral testing was performed at 2, 8 and 14 days post-stroke. Animals receiving AAV-GLT1, compared to AAV-GFP, showed significant decreases in the duration and magnitude of extracellular glutamate, measured by microdialysis, during the 60 minute MCAo. A significant reduction in brain infarction and DNA fragmentation was observed in the region of AAV-GLT1 injection. Animals that received AAV-GLT1 showed significant improvement in behavioral recovery following stroke compared to the AAV-GFP group. We demonstrate that focal overexpression of the glutamate transporter, GLT-1, significantly reduces ischemia-induced glutamate overflow, decreases cell death and improves behavioral recovery. These data further support the role of glutamate in the pathogenesis of ischemic damage in brain and demonstrate that targeted gene delivery to decrease the ischemia-induced glutamate overflow reduces the cellular and behavioral deficits caused by stroke.

## Introduction

An ischemic stroke begins with obstruction of an arterial vessel in the brain, progresses through a cascade of cellular and molecular events, and ultimately leads to cell death. The initial depletion of oxygen leads to a loss of cellular ATP and dysregulation of ion homeostasis at the membrane [1]. The altered ion homeostasis activates voltage-dependent calcium channels and the depolarization of the neuronal membrane can cause massive release of neurotransmitters such as glutamate. For glutamatergic neurons, ischemia causes the release of glutamate into the synaptic cleft and extracellular space. The excess glutamate in the extracellular space causes extended activation of ionotropic glutamate receptors (iGluRs). The resulting overstimulation of iGluRs activates intracellular signaling cascades producing excitotoxicity and cell death [2]. The inhibition of glutamate-induced excitotoxicity has been a therapeutic target for the treatment of stroke for many years [3]. For example, acute administration of NMDAR or AMPAR antagonists reduces ischemic damage in rodent models of stroke [4], [5]. Our lab has also previously reported that blocking glutamate release or glutamate-mediated post-synaptic excitability reduces neural degeneration in stroke rats [6], [7]. Taken together, these data support that regulation of glutamate overflow during the ischemic phase can alter outcomes in stroke animals, however, clinical trials based on iGluR antagonists have failed with adverse CNS effects and they possibly impede endogenous neurorepair mechanisms [8].

The release of glutamate occurs within minutes of ischemic onset and therapeutic drugs targeted at blocking excitotoxicity must be administered rapidly or they lose their protective effect. An alternative approach is to augment glutamate clearance from the extracellular space to prevent excitotoxic iGluR stimulation. To date, there are five subtypes of the sodium-dependent excitatory amino acid transporters (EAAT1-5) that differ in their regional and cellular expression and ability to transport glutamate [9]. Transient middle cerebral artery occlusion (MCAo) decreases GLT-1 (EAAT2), a predominantly glia-expressed EAAT [10], [11]. Furthermore, decreasing GLT1 mRNA using anti-sense knockdown exacerbated neuronal death caused by transient MCAo [12]. Certain small molecules such as beta-lactams have been shown to increase the expression of the glutamate transporter in the brain [13]. For example, Chu et al. demonstrated that intraperitoneal administration of ceftriaxone (CTX) for five consecutive days prior to stroke reduced infarction volume and increased behavioral recovery [14], [15]. To further explore this treatment strategy of stroke by increasing glutamate clearance, we generated an adeno-associated viral vector expressing the rat GLT-1 cDNA (AAV-GLT1). Our data show that intracortical delivery of AAV-GLT1 reduced ischemic damage and the magnitude and duration of ischemia-induced glutamate overflow caused by MCAo.

## Materials and Methods

### Animals

Adult male Fischer 344 rats (Harlan Laboratories Inc., IN) and Sprague-Dawley rats (Charles River Laboratory, NC) were used for amperometry and for MCAo studies, respectively and maintained under a 12-hour light–dark cycle. Housing and experimental procedures followed the guidelines of the “Principles of Laboratory Care” (NIH publication No. 86-23, 1996) and experimental procedures were approved by the NIDA Animal Care and Use Committee (Animal Study Protocol#08-CNRB-73) and the U. Kentucky Institutional Animal Care and Use Committee (Animal Study Protocol#880M2005).

### Construction, Packaging, Purification, and Titering of AAV Vectors

The rat cDNA for GLT-1 was first PCR amplified from pc-GLT-1 [16] (from Dr. Mike Robinson at U. Pennsylvania). The amplified PCR product contained BamHI (5′ end) and XbaI (3′ end) restriction enzyme sites used to insert the PCR product into pCR8/GW/TOPO (Invitrogen, CA) as an intermediate. The BamHI/Xba1 fragment replaced GFP in the BamHI/XbaI digestion of pssAAV-GFP [17] to create pssAAV-GLT1. Viral stocks of AAV-GLT1 serotype 1 were prepared using the triple-transfection method [17], [18], [19].

### *In vitro* characterization

HEK293 cells (American Type Culture Collection,Manassas VA) were transfected with AAV vector packaging plasmids containing GFP or GLT-1 cDNAs using Lipofectamine 2000 (Invitrogen). Western blot analysis was performed as previously described [20] using a mouse monoclonal anti-GLT1 antibody (from Dr. Jeffrey Rothstein, Johns Hopkins University) [21], [22]. Experiments were conducted twice with three wells of 24 well plate per group.

### Intracerebral AAV injections

For *in vivo* characterization by amperometry in control rats, animals were anesthetized with isoflurane. AAV-GLT1 or AAV-GFP/RFP (3 ul) was stereotaxically injection into the striatum (AP: +1.0, ML: 2.5, DV: −4.5 mm).

*For MCAo experiments:* Animals were anesthetized with chloral hydrate (0.4 g/kg, i.p.). AAV-GFP or AAV-GLT1 (2 ul per site; titer ∼10^13^ viral genomes/ml) was given intracerebrally into three cortical sites in the distribution of the right MCA [20].

### Quantification of extracellular glutamate by amperometry

Three weeks after viral infusion, rats were anesthetized with urethane (1.25 g/kg, i.p.). Ceramic-based microelectrode arrays (MEAs) consisting of four platinum recording sites (15×333 µm) specific for glutamate were used for recordings [23]. Glutamate (1 mM, 25–350 nL) was locally applied through a glass micropipette. Glutamate clearance rate was examined at 0.1 mm lateral to the infusion site (AP: +1.0, ML: 2.6 mm, DV: −4.5, mm).

### Quantification of glutamate release during MCAo using microdialysis and HPLC

The probe was stereotaxically placed in the parietal cerebral cortex (−4.0 mm posterior, 4.5 mm lateral to the bregma, −3.0 mm below brain surface). Artificial cerebrospinal fluid (aCSF) was perfused through the probe at a rate of 2×10^−6^ L/min. Samples were collected at 20 min intervals. The concentration of glutamate in the dialysis samples was determined using HPLC-based methods [24].

### Middle cerebral artery occlusion (MCAo)

Three weeks after the AAV injections, animals were anesthetized with chloral hydrate (0.4 g/kg, i.p.). The right MCA was transiently ligated with 10-O suture for 60-minute [25]. Core body temperature was maintained at 37°C with a heating pad during surgery. After surgery, the animals were kept in a temperature-controlled incubator to maintained body temperature at 37°C.

### Triphenyltetrazolium chloride (TTC) staining

Animals were sacrificed 2 days after MCAo. The brain slices (2 mm) were incubated in 20 g/L TTC [7].

### Behavioral recovery

At 2, 8 and 14 days after stroke, the elevated body swing test for body asymmetry and Bederson's test for neurological deficits were performed as previously described [20], [26], [27].

### Histological analysis

Rats were decapitated 1 day after MCAo or 3 weeks after AAV-GFP injection for TUNEL labeling as previously described [7]. Histological images taken from 0.05 mm slices were acquired using Nikon Super Coolscan table scanner and pixel density of TUNEL-positive nuclei per measured area of cortex was quantified and averaged from three sections corresponding to regions of TTC slices 3, 4 and 5. The TUNEL pixels density was measured using NIS Elements 2.3 software.

### Statistical analysis

Student's t-test, (two-tailed) was used for statistical analysis between two groups. For 2 factors (i.e. virus and time or virus and tissue slice number) a 2-way ANOVA with Bonferonni post-hoc analysis was used. Data are presented as mean ± S.E.M.

## Results

### AAV-GLT1 characterization and distribution of viral gene expression *in vivo*

**Figure 1:**
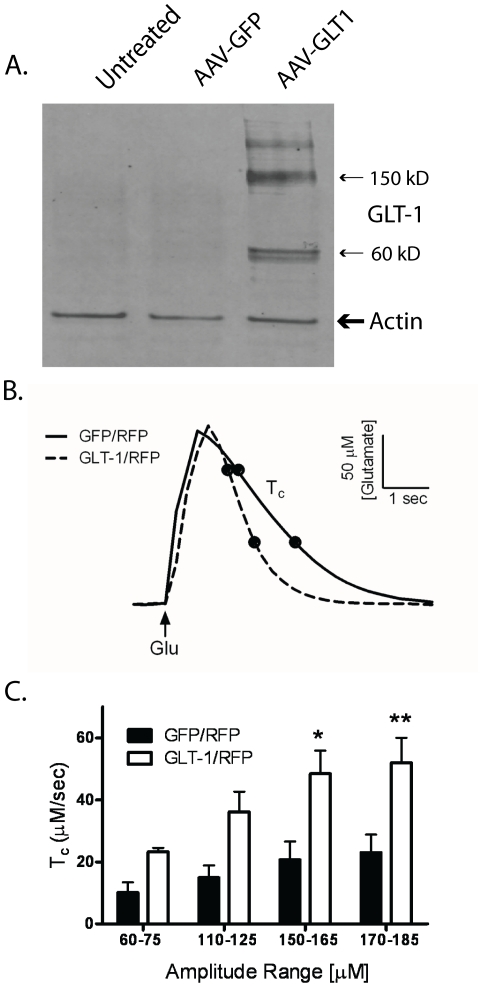
Characterization of AAV-GLT1 *in vitro* and *in vivo*. (A) Western blot analysis for GLT-1 protein in HEK293 cells that were transfected with the AAV-GLT1 packaging plasmid for 24 hours. GLT-1 appears as two prominent bands 150 kD and 60 kD [21], [22]. (B) Representative graphs showing time course of glutamate signals (amplitudes in 170–185 uM glutamate range) and the faster glutamate clearance rate in the striatum of animals injected with AAV-GLT1 compared to AAV-GFP. (C) Glutamate clearance (Tc) in the striatal hemisphere treated with AAV-GLT1 was significantly increased compared to the AAV-GFP -treated hemisphere. * p<0.05, ** p<0.01.

The rat cDNA for the glutamate transporter 1 was successfully subcloned into an AAV vector to generate pssAAV-GLT1 as determined by DNA sequencing (data not shown). Expression of the rat GLT1 protein was confirmed by western blot analysis of protein extracts prepared from HEK293 cells transfected with the pAAV-GLT1 packing plasmid into HEK293 cells (Figure 1A). Primary cortical neurons transduced with AAV-GLT1 virus exhibited increased GLT1 immunoreactivity that was localized throughout the cell (Figure S1). Biochemical function of GLT-1 protein was verified *in vivo* using glutamate specific electrodes and amperometry in adult rat brain three weeks after AAV-GLT1 injections (Figure 1B and 1C). The glutamate clearance (Tc) was significantly faster in striata injected with AAV-GLT1 (n = 3 rats) compared to those injected with AAV-GFP (n = 3 rats), (F(1,16) = 31.97, p<0.0001, n = 3; Figure 1C.

**Figure 2:**
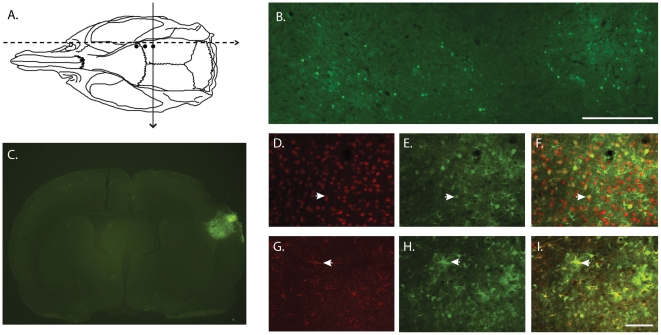
Transduction of rat cortex by three site injection of AAV-GFP serotype 1. (A) Schematic representation of rat skull with injection sites (black dots). (B) Dashed line points to brain section cut in a sagittal plane showing 3 injections sites by GFP fluorescence (green;scale = 0.5 mm). (C) Coronal section of injection site showing expression of GFP from a dsAAV1eGFP injection. Coronal sections immunostained for neuronal marker, NeuN (red; D–F) or the astrocytic marker, GFAP (red;G–I) show that GFP (green) colocalizes to both neurons and astrocytes in the cortex. Arrowheads illustrate example of NeuN (D,E) or GFAP (G,H) double-labeled cells. Scale bar = 100 um.

The distribution of viral transduction by AAV serotype 1 was examined at three weeks after intracortical AAV-GFP injection (n = 3 rats). There is a widespread distribution of GFP fluorescence in cortical region which colocalizes to both neurons (NeuN immunopositive) and astrocytes (GFAP immunopositive; Figure 2).

### AAV-GLT1 reduces infarction and improves behavioral recovery

**Figure 3:**
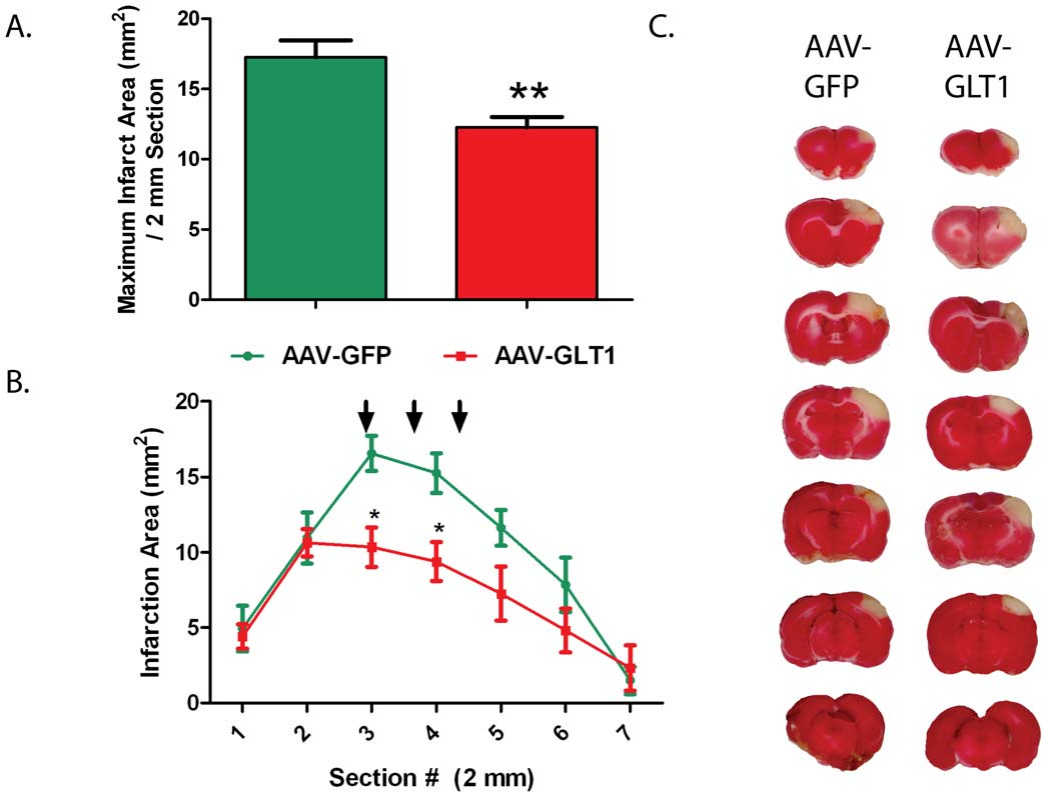
AAV-GLT1 significantly reduces infarction size in region of viral injection. Local administration of AAV-GLT1 reduced brain infarction. (A) AAV-GLT1 significantly reduced the maximal infarct area compared to AAV-GFP treated animals (p<0.01, Student's t-test). (B) Analysis of infarction based on 2 mm brain section showed a significant reduction in brain infarction in animals treated with AAV-GLT1 (p<0.01, Two-way ANOVA). Significant differences in the infarction area per slice were observed in slices 3 through 5 (arrows) which contain the viral injection sites (*p<0.05). (C) Representative 2 mm brain sections stained with TTC. Rats received 3 cortical injections of AAV-GLT1 or AAV-GFP into the right hemisphere.

Cerebral infarction was examined by TTC at 2 days after MCAo. Pretreatment with AAV-GLT1 (n = 7 rats) significantly reduced infarction compared to AAV-GFP injections (n = 8 rats; Figure 3A, p<0.01, Student's t-test and Figure 3C, p<0.05, 2-way ANOVA). Post-hoc analysis indicates that the significant reduction of infarction by AAV-GLT1 occurred on brain sections containing the cortical region corresponding to the site of intracortical injections of AAV-GLT1 (Figure 3B, p<0.05, Bonferroni post-test).

**Figure 4:**
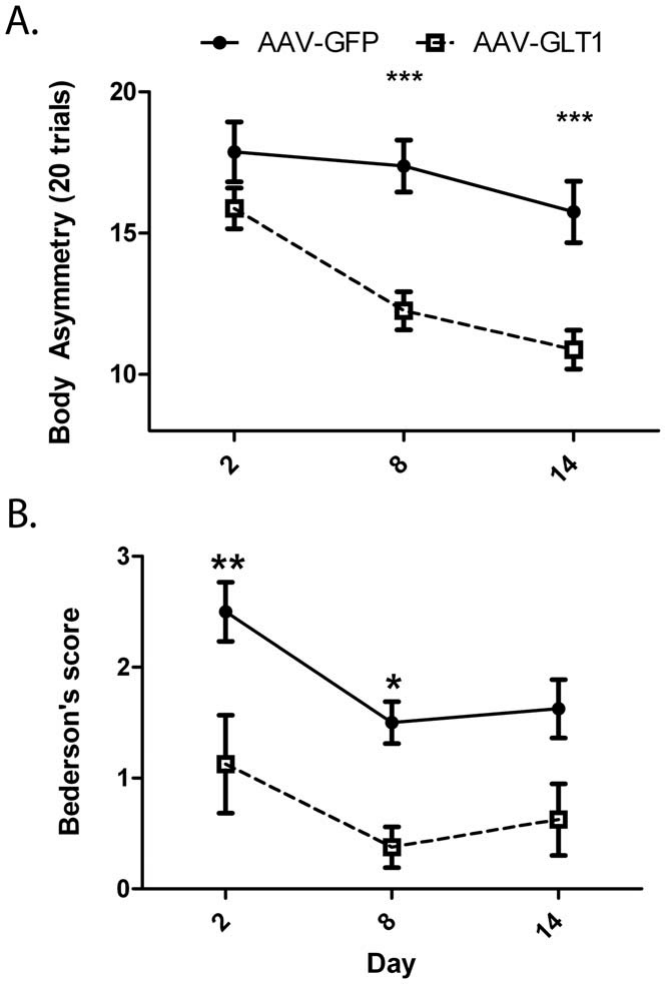
AAV-GLT1 promotes behavioral recovery after cerebral ischemia. Behavioral tests were carried out at 2, 8 and 14 days after the MCAo and analyzed with 2-way ANOVA. AAV-GLT1 injection significantly reduced body asymmetry in an elevated body swing test (A) and neurological abnormality scores in Bederson's test (B). *p<0.05, **p<0.01, ***p<0.001; Bonferroni test.

Functional recovery was examined at 2, 8 and 14 days after the MCAo in 16 rats. AAV-GLT1 (n = 8 rats) injection significantly reduced body asymmetry in an elevated body swing test (Fig. 4A, p<0.0001, 2-way ANOVA ) and Bederson's neurological abnormality scores (Fig. 4B, p<0.001, 2-way ANOVA). Prior to MCAo surgery, animals showed no significant difference in baseline locomotor activity for any parameters (i.e. horizontal activity, vertical activity, total distance or stereotypy (data not shown). No significant difference in locomotor activity was observed at 24 hours post-stroke between AAV-GLT1 and AAV-GFP treated animals (data not shown).

Physiological parameters were measured before and 30 minute after MCAo. We found no significant difference in blood pH, blood pressure, temperature, hematocrit% or blood gas levels between AAV-GFP and AAV-GLT1 treated animals (Table 1). These data suggest the protective effects of AAV-GLT1 were not due to systemic changes.

**Table 1:** GLT1 over-expression did not alter physiological parameters 30 min after MCAo.

	GFP	GLT1	* p
pH	7.44±0.01	7.42±0.03	0.57
PaCO_2_ mmHg	37.8±2.3	41.8±3.4	0.36
PaO_2_ mmHg	98.3±5.3	93.3±6.9	0.59
MBP mmHg	99.5±4.2	96.0±9.6	0.75
Hematocrit %	37.8±1.5	33.0±2.4	0.15

### AAV-GLT1 decreases ischemia-induced glutamate overflow in lesioned cortex

**Figure 5:**
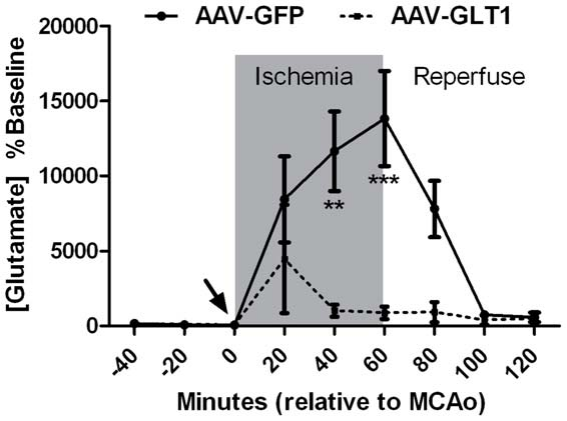
Pre-treatment with AAV-GLT1 reduces glutamate overflow in stroke rats. Microdialysis was performed in the lesioned cortex. In animals receiving AAV-GFP, MCAo caused an increase in extracellular glutamate. In animals receiving AAV-GLT1, MCAo -induced glutamate overflow was significantly reduced (p<0.01, Two-way, AAV-GLT1 vs AAV-GFP post-MCAO;*p<0.05, **p<0.01, ***p<0.001; Bonferroni test.

To determine whether the overexpression of GLT1 reduced glutamate efflux caused by ischemia, we performed *in vivo* microdialysis to sample extracellular fluid in the cortical region corresponding to the AAV-GLT1 or AAV-GFP injection prior, during and after the 60 minute MCAo in 8 rats. An increase in extracellular glutamate was detected at 20 minutes after MCAo initiation for both AAV-GFP (n = 4 rats) and AAV-GLT1 (n = 4 rats) treated animals (Figure 5). Glutamate remained elevated until 20 minutes after the 60 minute MCA occlusion in the AAV-GFP treated animals. A significant reduction in ischemia-induced glutamate overflow was found in animals receiving AAV-GLT1 (p = <0.0001, 2-way ANOVA). Glutamate levels returned to baseline by 40 minutes after MCAo initiation in the AAV-GLT1-treated animals (Figure 5).

### MCAo-related TUNEL was reduced by AAV-GLT1

**Figure 6:**
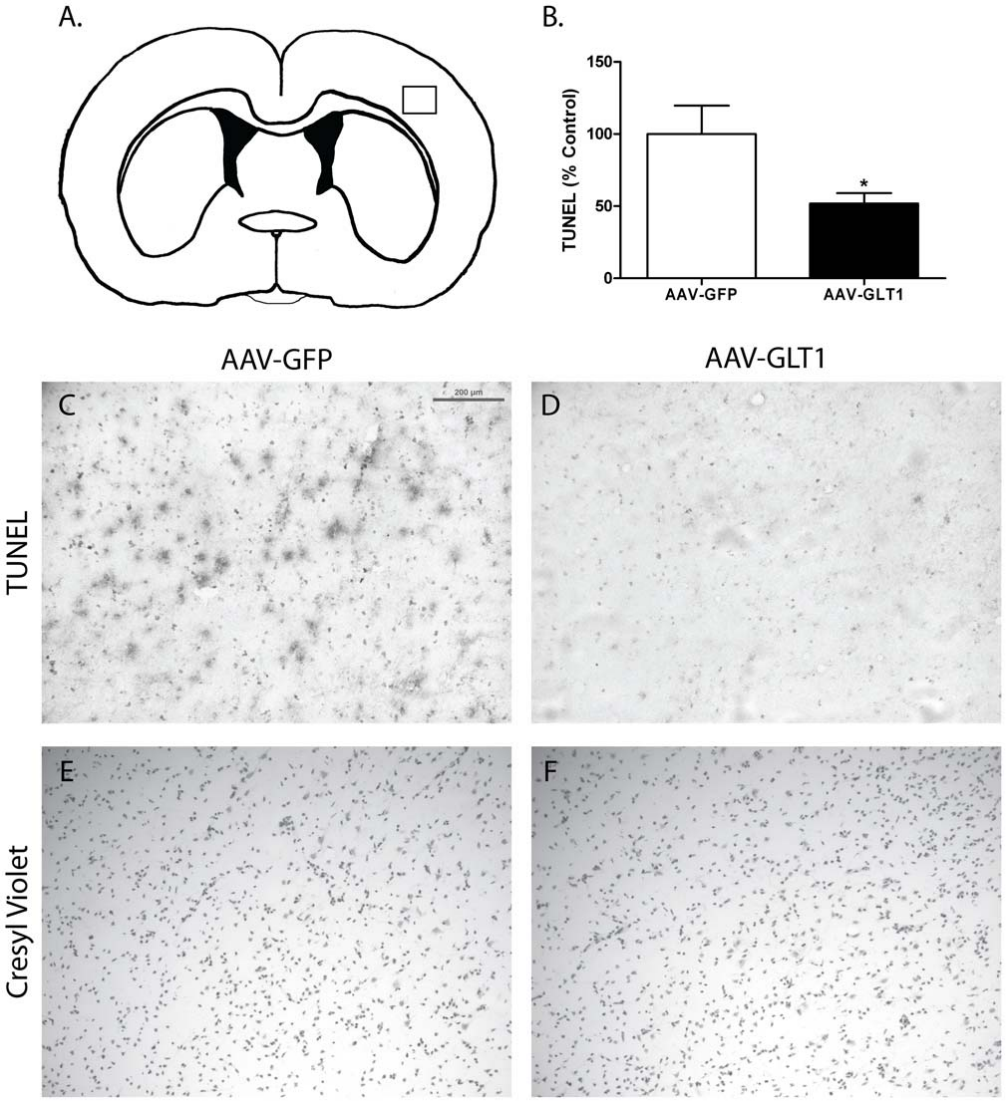
Pre-treatment with AAV-GLT1 reduces TUNEL staining following MCAo. TUNEL was examined at 24 hours after MCAo in the region of infarction near site of viral transduction. (A) Schematic of coronal secion near bregma indicating cortical region (box) of representative images in C–F. (B) AAV-GLT1 significantly reduced TUNEL staining in ischemic cortex compared to AAV-GFP (p<0.05, Student's t-test). Representative TUNEL from AAV-GFP (C) and AAV-GLT1(D). Cresyl violet staining in on adjacent brain sections from AAV-GFP (E) and AAV-GLT1(F) treated animals. Scale bar = 0.2 mm.

TUNEL was examined in the lesioned cortical region at 24 hours after the onset of ischemia in 14 rats. Animals that received pretreatment with AAV-GLT1 (n = 7 rats) showed a significant (48%) reduction in TUNEL staining compared to the AAV-GFP (n = 7 rats) treated animals (Figure 6, p<0.05, Student's t-test). These data further support that AAV-GLT1 conferred a protective effect against MCAo-induced ischemia.

## Discussion

In the current study, we successfully generated and characterized an AAV vector to express rat GLT-1 cDNA (AAV-GLT1). Using AAV-GLT1, we demonstrated that over-expression of GLT1 in rat parietotemporal cortex 3 weeks prior to MCAo reduced infarction caused by MCAo, improved neurological deficits, decreased the magnitude and duration of ischemia-induced glutamate overflow and attenuated ischemia-related TUNEL staining. Collectively, our data indicated that increasing the capacity to clear extracellular glutamate via gene transfer provides beneficial outcomes against ischemia-induced glutamate release and associated excitotoxicity.

The overflow of glutamate following ischemia has been known for over 25 years [28]. Excess extracellular glutamate causes increased activation of ionotropic GluRs such as NMDA and, to a lesser extent, AMPA. The increased influx of calcium due to the activation of post-synaptic iGluRs initiates a range of neurotoxic cascades [2]. Studies have shown that acute administration of the NMDAR or AMPAR antagonists reduce ischemic damage in rodent models of stroke [4], [5]. Our lab also reported that pharmacological agents that block glutamate-mediated post-synaptic excitability or glutamate release can reduce neural degeneration in stroke rats [6], [7]. Our current study confirms the significant contribution of ischemia-induced glutamate overflow to the pathogenesis of stroke. Additionally, our study extends the pharmacological manipulation of GLT1 levels [13], [14], [15] to genetic manipulation of GLT1 as a gene therapy approach to reducing ischemia-induced excitotoxicity. Here, we used an AAV vector to deliver the rat GLT1 cDNA to rat cortex prior to ischemia and showed that the duration and magnitude of ischemia-induced glutamate overflow was significantly reduced. We observed a significant reduction in the amount of infarcted tissue and ischemia-induced TUNEL staining in the region of tissue transduced with AAV-GLT1. These histological findings support that augmenting GLT1 expression is protective against the ischemic damage. There was also a progressive improvement in the recovery of neurological deficits (Bederson's score) and body asymmetry in animals that received AAV-GLT1 but not the control virus AAV-GFP. Collectively, the behavioral data also suggest that overexpression of AAV-GLT1 at the time of ischemia can reduce the ischemia-induced glutamate overflow and confer protection against excitotoxicity.

Endogenous GLT1 is primarily localized to perisynaptic membranes of astrocytes [29]. By using an AAV serotype 1 which transduces both neurons and glia in rat cortex [17], we are able to target GLT1 on both neuronal and astrocytic membranes. In primary cortical neurons, we observed GLT1 expression distributed throughout GLT1-immunoreactive cells. Using our 3 site injection paradigm of AAV1-GFP, we observed widespread distribution pattern of GFP that colocalized to both neurons (NeuN-positive) and astrocytes (GFAP-positive cells). These data support that the overexpression of GLT1 is widely distributed with respect to the cellular elements that would transport elevated glutamate following ischemia.

We, and others, previously reported that pretreatment with GluR antagonists reduces ischemic brain damages in experimental animals [30]. On the other hand, we also found excitatory amino acids have trophic response to developing neurons [31], [32]. Furthermore, systemic administration of the N-methyl-D-aspartate (NMDA) receptor blocker MK-801 suppressed the elevated neurogenesis in hippocampus after MCAo in rats [33]. These data support two opposite reactions of GluR antagonists in stroke. Systemic application of GluR antagonists reduces neurodegeneration in the ischemic region, but attenuates neuroregeneration in non-injured regions. These conflicting responses may result in the failure in clinical trials of using GluR antagonists in stroke patients [34]. A proper approach would be reducing glutamate concentrations or responses locally in the lesioned area while preserving the endogenous glutamate function in areas for neurogenesis, such as subventricular zone [35]. Such an approach was shown in this study where overexpression of GLT1 in the ischemic cortex reduces the size of lesion and improves the behavioral recovery up to two weeks after stroke.

The experimental approach used in this study evaluated glutamate clearance following ischemia-induced overflow where the glutamate transporter had been genetically manipulated to be overexpressed in the region of ischemia. The findings of reduced glutamate overflow and reduced ischemic damage further support strategies aimed at reducing the duration and magnitude of extracellular glutamate caused by an ischemic event. Although clinical trials targeted at systemic glutamate suppression failed, the current study suggests that focal glutamate clearance may be beneficial. For example, people at risk for transient ischemic attacks, previous surgery, heart attack or other non-preventable risk factors for stroke may benefit from elevated GLT-1 levels caused by focal pharmacological [13], [14], [15] or genetic manipulation as less invasive and selective delivery of genes to the brain emerges.

### Summary

In conclusion, we used a viral vector-mediated gene delivery approach to increase the expression of GLT-1 locally and reduce the damage caused by brain ischemia in a rodent model of stroke. Our data support approaches to selectively upregulate endogenous GLT-1 in the cortex for the protection against stroke [13], [14], [15]. Further development of selective gene delivery may make the use of GLT1 a more viable gene therapy approach for neurodegeneration caused by glutamate excitotoxicity.

## Supporting Information

Figure S1 **GLT-1 immunostaining (red) of rat primary cortical neurons nine days after transduction with control virus (AAV-GFP) or AAV-GLT1.** Nuclei stained with DAPI (blue). Primary cortical neurons from E15 Sprague-Dawley rat embryos [19] were transduced with AAV-GFP or AAV-GLT1 on DIV6. Cells were fixed on DIV15 and were immunostained using rabbit polyclonal anti-GLT1 antibody using methods previously described [19]. Experiments were conducted on two independent primary culture preparations with 6 wells of 96 well plate per group.
(TIF)
