# Supplementary figures and images for: Targeted Over-Expression of Glutamate Transporter 1 (GLT-1) Reduces Ischemic Brain Injury in a Rat Model of Stroke
Source: PLoS One. 2011 Aug 10;6(8):e22135. doi: 10.1371/journal.pone.0022135 (PMC3154194; doi:10.1371/journal.pone.0022135)

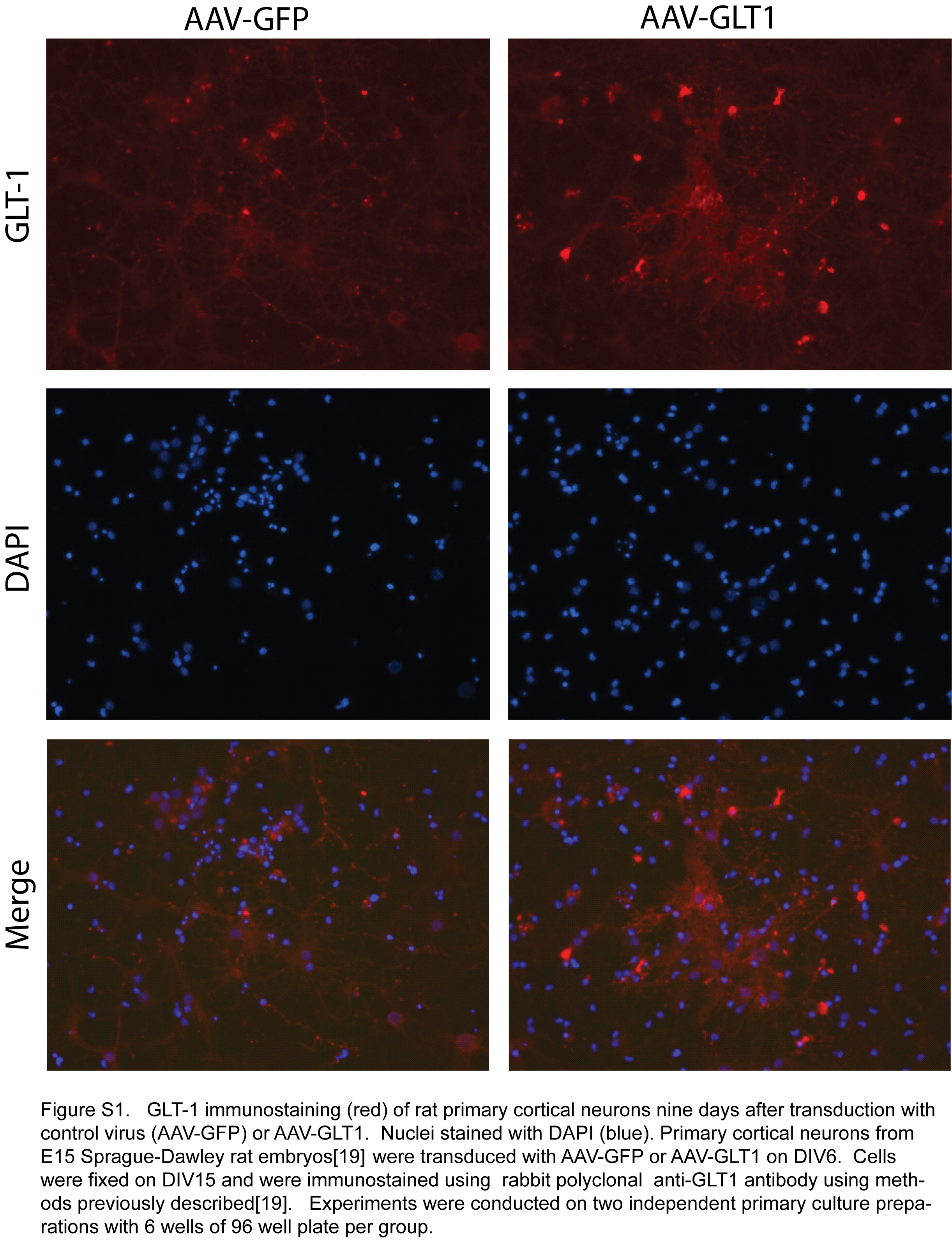

Supplement: Figure S1 — GLT-1 immunostaining (red) of rat primary cortical neurons nine days after transduction with control virus (AAV-GFP) or AAV-GLT1. Nuclei stained with DAPI (blue). Primary cortical neurons from E15 Sprague-Dawley rat embryos [19] were transduced with AAV-GFP or AAV-GLT1 on DIV6. Cells were fixed on DIV15 and were immunostained using rabbit polyclonal anti-GLT1 antibody using methods previously described [19]. Experiments were conducted on two independent primary culture preparations with 6 wells of 96 well plate per group. (TIF) [file pone.0022135.s001.tif]
